# COLOSTRUM FAT AND ENERGY CONTENT: EFFECT OF GESTATIONAL AGE AND FETAL GROWTH

**DOI:** 10.1590/1984-0462/;2018;36;3;00006

**Published:** 2018

**Authors:** Luiza Tavares Carneiro Santiago, José Donizeti de Meira, Natália Alves de Freitas, Cilmery Suemi Kurokawa, Lígia Maria Suppo de Souza Rugolo

**Affiliations:** a Universidade Estadual Paulista, Botucatu, SP, Brasil.

**Keywords:** Infant, Premature, Breast feeding, Colostrum, Fat, Recém-nascido, Prematuro, Aleitamento materno, Colostro, gordura

## Abstract

**Objective:**

To determine whether fat content and energy value change in colostrum according to gestational age and fetal growth.

**Methods:**

Cross-sectional study with mothers of preterm and term infants born in a tertiary center in 2015-2016. Inclusion criteria: single pregnancy, absence of diabetes, chorioamnionitis and mastitis, no use of illicit drugs or alcohol, without fetal congenital malformation or infection. Four groups were formed according to gestational age and fetal growth: preterm infants small for gestational age (PT-SGA; n=33) and appropriate for gestational age (PT-AGA; n=60), term infants small for gestational age (T-SGA; n=59) and appropriate for gestational age (T-AGA; control, n=73). Colostrum was collected between 24-72 hours postpartum. Gestational and birth variables were analyzed. Outcome variables were: fat content in colostrum (evaluated by crematocrit method) and estimated energy value. Chi-square or Fisher exact tests, ANOVA, and multivariable linear regression were used for comparison among groups.

**Results:**

Mean gestational age was 34 weeks in preterm infants and 39 weeks in term neonates. Crematocrit did not differ between groups, with mean values varying between 3.3 and 4.0%; estimated energy value was 52 to 56 kcal/dL. Crematocrit ≥4% was more frequent in the T-SGA group. Only in the PT-SGA group there was a correlation between crematocrit and body mass index of the mother.

**Conclusions:**

The fat content and energy value of colostrum did not change according to gestational age or fetal growth.

## RESUMO

**Objetivo:**

Avaliar se o conteúdo de gordura e o valor energético estimado no colostro diferem em função da idade gestacional e do crescimento fetal.

**Métodos:**

Estudo transversal com mães de recém-nascidos pré-termo e a termo nascidos em centro terciário em 2015-2016. Critério de inclusão: gestação única, sem diabetes, corioamnionite e mastite, sem uso de drogas ilícitas e álcool, ausência de malformação ou infecção congênita fetal. Foram constituídos quatro grupos conforme idade gestacional e crescimento fetal: pré-termo pequeno para a idade gestacional (n=33); pré-termo adequado (n=60); a termo pequeno (n=59) e a termo adequado para a idade gestacional (controle, n=73). O colostro foi coletado por extração manual entre 24 e 72 h pós-parto. Foram analisadas variáveis gestacionais e de nascimento. Os desfechos foram o conteúdo de gordura no colostro, pelo método do crematócrito, e o valor energético estimado. Na comparação entre grupos foram utilizados os testes do qui-quadrado ou Exato de Fisher, ANOVA e regressão linear multivariada.

**Resultados:**

A idade gestacional média foi de 34 semanas nos neonatos pré-termo e 39 semanas nos a termo. O crematócrito não diferiu entre os grupos, com valores médios de 3,3 a 4,0%; e o valor energético estimado foi de 52 a 56 kcal/dL. Crematócrito ≥4% foi mais frequente no grupo a termo pequeno para idade gestacional. Apenas no grupo de recém-nascidos pré-termo pequenos para a idade gestacional houve correlação entre crematócrito e índice de massa corpórea materno.

**Conclusões:**

O conteúdo de gordura e o valor energético estimado do colostro não diferiram em função da idade gestacional e do crescimento fetal.

## INTRODUCTION

Breastfeeding is considered the gold standard of infant nutrition and has been strongly encouraged for preterm newborns due to the immunological properties of breast milk, its role in gastrointestinal maturation and in the establishment of mother-child bond, thus contributing to a better growth and development prognosis.^1^ ^,^ ^2^ ^,^ ^3^ ^,^ ^4^

Lactation is classically known to have three phases, the first one being represented by colostrum, produced in the first five days after delivery. Colostrum is a small amount of fluid that is rich in immunological components, lactoferrin, leukocytes and growth factors, and which has relatively low concentrations of lactose and higher protein and lipid content compared to mature milk.^5^ ^,^ ^6^ The second phase is transitional and occurs from the sixth day until the end of second week after delivery; only then is the milk classified as mature.^7^

Breastmilk is an adequate combination of macronutrients (proteins, lipids and carbohydrates) and micronutrients, including minerals, vitamins and various bioactive components.^8^ ^,^ ^9^ The difference in milk composition of pre-term infants’ mothers is already well documented in the literature as compared to mothers of full-term newborns, with higher protein and lipid content, and consequent higher energy value in the breast milk for preterm infants.^9^ In the first eight weeks of lactation, mean values reported are: 1.3 g/dL of protein; 3.5 g/dL of lipid; and 7.3 g/dL of carbohydrates in the milk of preterm neonates’ mothers. However, these data should be interpreted with caution, since these values refer to the average concentrations found in colostrum and mature milk.^10^

Specifically on lipids, their concentration in breast milk differs according to geographic conditions, mothers’ eating habits, gestational and postnatal age, period of the day, maternal nutritional status, and breastfeeding time (greater quantity at the end of feeding).^11^ ^,^ ^12^ Significant differences in breast milk lipid content are described as related to infants born under 30 weeks, with mean values of 4.0 g/dL by one week of lactation, while values related to infants born after 30 weeks and to term are similar (mean 2.5 g/dL).^2^ This difference in lipid composition of the milk of preterm infants mothers’ deserves attention, since lipids are essential not only for energy supply, but also for the development of the child’s central nervous system and retina.^13^

Despite the existence of several studies on breast milk, data on the effects of fetal growth on the composition of breast milk are scarce, as well as studies investigating the effects of gestational age and fetal growth on colostrum composition. The current spotlight on colostrum use increases the interest and the need to deepen knowledge about its composition and possible variations according to gestational age and fetal growth. Thus, the objective of this study was to investigate whether the fat content and energy value of colostrum change as per gestational age and fetal growth.

## METHOD

Cross-sectional study involving puerperal women who gave birth at the Maternity Hospital of the Botucatu Medical School (Universidade Estadual Paulista - UNESP), in 2015-2016. The research project was approved by the Research Ethics Committee of the institution and all participants signed the informed consent form.

Participants were postpartum women who met the following criteria: single gestation; absence of diabetes and chorioamnionitis; no use of illicit drugs and alcohol; absence of fetal malformation and congenital infection. Mothers with mastitis and who were unable to withdraw the necessary minimum volume of colostrum were not included.

The sample had the maximum number of puerperal women who met the inclusion criteria in the study period. In total, 225 mothers were assessed and distributed in the study groups, with test power greater than 80%.

Four study groups were assembled based on gestational age and fetal growth: preterm infants small for gestational age (PT-SGA); preterm neonates appropriate for gestational age (PT-AGA); term infants small for gestational age (T-SGA) and term newborns appropriate for gestational age (T-AGA or control).

Gestational age was calculated by the best obstetric estimate (first trimester ultrasound or precise date of last menstrual period). Adequacy of birth weight to gestational age was based on intrauterine growth chart by Fenton and Kim,^14^ being classified as appropriate for gestational age when birth weight was between the 10^th^ and 90^th^ percentile of the growth curve, and small for gestational age when the weight was below the 10^th^ percentile.

Independent variables were: maternal data such as age, schooling, parity, smoking habit, pre-gestational body mass index (BMI), premature rupture of membranes, fetal distress, and type of delivery; and newborn data such as gestational age, need for resuscitation at birth, 1-minute APGAR score, birth weight and appropriateness of weight for gestational age. Outcomes of interest were fat content and estimated energy value of colostrum in all four study groups. Correlation between crematocrit and maternal BMI were defined as secondary outcomes.

Samples of 0.5 to 1.0 mL of colostrum were collected by manual extraction between 24 and 72 hours after delivery, in the morning and in the feeding interval. The fat content was estimated by the crematocrit method, as described by Lucas et al.^15^ This is a simple, inexpensive and reproducible method, with coefficient of variation <1%.^16^ To determine the crematocrit, two capillary tubes were filled with colostrum immediately after collection, one of the ends being sealed and the tubes centrifuged for 15 minutes at 12,000 rpm in a microcentrifuge (Celm^®^). Crematocrit was expressed by the percentage of cream column in the capillary tube with a cremometer (millimeter ruler). It was every time measured by the same author, who was blinded to the group each sample belonged to. The energy value of colostrum samples was calculated as proposed by Lucas et al.^25 (^ ^Equation 1^ ^)^:

Energy (kcal/dL) = 5.99 x crematocrit (%) + 32.5. (1)

Continuous variables are described in tables with respective mean and standard deviations, and categorical variables are expressed as number and proportion of events. In the study of associations between groups, chi-square or Fisher exact tests were used for categorical variables and ANOVA for continuous variables. The effect of potential confounding factors on outcomes was controlled in a multiple linear regression model, which included all variables that, in the univariate analysis, differed significantly between groups. Pearson’s multiple correlation was used to estimate the power of the association between crematocrit and maternal BMI. In all analyses, significance level was set at 5%.

## RESULTS

From August 2015 to July 2016, 275 postpartum women meeting our inclusion criteria were selected; however, 50 were excluded because they failed to obtain the required volume of colostrum. Thus, the samples of 225 puerperal women were studied and divided into four groups: PT-SGA (n=33); PT-AGA (n=60); T-SGA (n=59); and T-AGA (n=73).

Among puerperal women studied, more than 90% had prenatal care and 19% were smokers, with no differences between groups. The main maternal and gestational data are presented in Table 1, which shows that the maternal BMI was higher in the T-AGA group compared to all others, as well as the percentage of overweight/obesity, although it was not statistically significant. Fetal distress and C-section delivery were more frequent in the PT-SGA group (Table 1).

**Table 1:** Maternal and gestational data of all groups.

	PT-SGA (n=33)	PT-AGA (n=60)	T-SGA (n=59)	T-AGA (n=73)	p-value
Age*	25±6	25±6	23±6	25±6	0.353
Adolescents (%)	27	27	29	12	0.083^a^
Primiparity (%)	49	32	49	26	0.018^a^
>8 years of education (%)	61	58	64	56	0.807^a^
Mother’s BMI*	24.7±7.6	24.7±5.7	23.3±5.3	26.8±5.8	0.011
Overweight/obesity (%)	44	37	32	53	0.102^a^
Premature rupture of membranes >18 h (%)	6	18	5	5	0.029^b^
Fetal distress (%)	27	7	3	4	<0.001^b^
C-section (%)	67	25	10	38	<0.001^a^

PT-SGA: preterm newborns, small for gestational age; PT-AGA: preterm newborns, adequate for gestational age; T- SGA: term newborns, small for gestational age; T-AGA: term newborns, adequate for gestational age (control); *mean±standard deviation (ANOVA followed by Tukey test (T-AGA> T-SGA); ^a^chi-square test; ^b^Fisher’s Exact test; BMI: body mass index.

Birth data are presented in Table 2 and show greater need for resuscitation and lower frequency of breastfeeding in the first hour of life in preterm birth groups. Mean gestational age in preterm groups was 34 weeks, which characterizes them as late preterm infants (Table 2).

**Table 2:** Birth data in all groups.

	PT-SGA (n=33)	PT-AGA (n=60)	T-SGA (n=59)	T-AGA (n=73)	p-value
Gestational age (weeks)*	34±2	34±3	39±1	39.5±2	<0.001
Birth weight (g)*	1760±395	2430±535	2640±230	3380±385	<0.001
Resuscitation upon birth (%)	21	18	7	1	0.001^b^
1-minute APGAR*	7±1	8±2	8±1	8±1	0.003
Breastfeeding in 1^st^ hour of life (%)	33	58	90	92	<0.001^a^

PT-SGA: preterm newborns, small for gestational age; PT-AGA: preterm newborns, adequate for gestational age; T- SGA: term newborns, small for gestational age; T-AGA: term newborns, adequate for gestational age (control); *mean±standard deviation (ANOVA followed by Tukey test); ^a^chi-square test; ^b^Fisher’s Exact test.

Table 3 shows that the mean values of crematocrit and estimated energy value did not differ between groups. However, higher values of crematocrit (≥4%) were predominant in the T-SGA group. Multiple linear regression analysis showed a correlation between crematocrit and maternal BMI only in the PT-SGA group (Table 3).

**Table 3:** Crematocrit, energy and correlation between crematocrit and mothers’ body mass index in all groups.

	PT-SGA (n=33)	PT-AGA (n=60)	T-SGA (n=59)	T-AGA (n=73)	p-value
Crematocrit (%)^a^	4.0±2.6	3.7±2.4	3.8±2.1	3.2±1.9	0.550
Crematocrit ≥4% (% samples)^b^	48.5	48.0	67.0	42.0	0.028
Energy (kcal/d)^a^	56.2±15.8	54.6±14.5	55.1±12.8	52.2±11.9	0.472
Crematocrit *versus* mother’s BMI^c^	r=0.620 (p<0.001)	r=0.100 (p=0.492)	r=-0.110 (p=0.382)	r=-0.005 (p=0.970)	

PT-SGA: preterm newborns, small for gestational age; PT-AGA: preterm newborns, adequate for gestational age; T- SGA: term newborns, small for gestational age; T-AGA: term newborns, adequate for gestational age (control); ^a^mean±standard deviation (ANOVA followed by Tukey test); ^b^chi-square test (contrast test: T-SGA>T-AGA=PT-AGA); ^c^Multivariate analysis including the following variables: parity, time of premature rupture of membranes, fetal distress, type of delivery, and mother’s body mass index; BMI: body mass index; r-Pearson’s correlation coefficient.

## DISCUSSION

This study showed that the fat content and the energy value of colostrum did not change according to gestational age and fetal growth. However, the results obtained in the SGA groups, whether with preterm or term newborns, suggest the existence of adaptive mechanisms in the mammary gland that aim to maintain the nutritional adequacy of breast milk to most vulnerable infants, that is, who did not have adequate fetal growth.

A common concern in preterm infant nutrition is the insufficient supply of energy, which can impair their growth. In this aspect, our study suggest that the energy supplied by the colostrum to preterm and SGA infants can be considered adequate, since it did not differ from that of the control group.

Lipids are the main energy source of breast milk, and assessing milk fat and energy content may help optimize the nutrition of preterm newborns. So the crematocrit method stands out as a simple and cheap way of estimating breast milk fat content and energy value that can be used in daily practice.^16^

Studies on breast milk crematocrit are scarce and have small samples. Weber et al.^17^ determined lipid content in the milk of 20 mothers of very low birth weight preterm infants according to circadian rhythm and lactation weeks. Mean values of lipid concentration in the first week were 3.9 g/dL, and variability throughout the day was: lower values in the morning (3.5 g/dL) and higher values in the afternoon and evening (approximately 4 g/dL).^17^ In the present study, samples were always obtained in the morning and results were similar.

Another study analyzed the lipid content and energy value of breast milk in the three stages of lactation, comparing 22 mothers of premature infants and 39 mothers of term newborns. In colostrum of mothers of preterm infants, crematocrit was lower than in that of term babies, although it was not statistically significant (4.9% *versus* 5.6%). Energy value did not vary significantly either. One limitation of this study was not reporting mean preterm gestational age, which ranged from 26 to 36 weeks.^18^

In Brazil, Grumach et al.^19^ investigated whether the macronutrient composition of milk from T-SGA mothers differs from that of PT-AGA and T-AGA (control) groups. Sixty-six puerperal women, divided into these three groups, were studied: 16 in the T-SGA, 20 in the PT-AGA, and 30 in T-AGA. Mean gestational age was 39 weeks among term infants and 34 weeks in preterm infants. The T-SGA group had lower crematocrit values in colostrum, while the PT-AGA group did not differ from control group. Variability in crematocrit was high, with colostrum values ranging from a minimum of 1% in all three groups to a maximum of 13% in control group. Median values were not presented,^19^ which makes it difficult to compare such results with those of our study.

Recent research has focused on the effect of fetal growth on maternal milk composition and on the role of lipids in the prognosis of neurodevelopment. Domany et al.^20^ investigated whether the fat content in breast milk changes according to fetal growth. Crematocrit was evaluated in the three stages of lactation of 26 mothers of newborns small for gestational age, compared to 30 mothers of newborns adequate for gestational weight. Mean values of crematocrit in three phases of lactation did not change between the two groups. However, interpretation of these results is limited by the sample not being stratified according to gestational age, which ranged from 25 to 41 weeks.^20^ Another study conducted with 60 puerperal women found no effect of fetal growth on the composition of milk fatty acids during the three phases of lactation, when controlled for gestational age.^21^

A recent study investigated the factors that influence the fatty acid profile in colostrum and reported that demographic aspects, including age and maternal nationality, had a greater influence compared to type of delivery and maternal BMI.^13^

The present study evaluated the effect of gestational age and fetal growth - as well as the possible influence of maternal, gestational, and birth factors - on the fat content of colostrum. Gestational age and fetal growth had no effect, only maternal BMI was correlated with crematocrit. A hypothesis for these results would be that, in the presence of adverse pregnancy situations, physiological changes occur in the lactation process to compensate intrauterine impairment and supply the newborn’s needs. Two important findings supported this hypothesis: first, although the mean crematocrit values ​​did not differ, the T-SGA group had a significantly higher percentage of colostrum samples with higher crematocrit value (≥4%). Also, the correlation between crematocrit and maternal BMI occurred only in the PT-SGA group, which suggests that the concomitant presence of both disorders, prematurity and inadequate fetal growth, could exceed the physiological limits of maternal compensation, making the colostrum fat content directly related to maternal nutritional status, which means greater vulnerability of this group.

This study has some limitations, though. In 18% of the eligible sample, the necessary volume of colostrum could not be obtained, predominantly after C-section delivery and when the newborn needed hospitalization in the Intensive Care Unit (ICU). Crematocrit was the method used due to its ease of execution, good reproducibility and low cost,^16^ ^,^ ^20^ however, there are more sensitive methods that allow to assess not only the amount but also the composition of lipids to be determined.^11^ ^,^ ^12^ The eating habits of puerperal women was not evaluated; the cross-sectional design of the study did not allow to analyze the changes in fat content according to the phases of lactation; the number of puerperal women in the PT-SGA group was relatively small, which did not allow subgroup analysis to further investigate the influence of maternal nutritional status on colostrum composition. However, in spite of the small number of PT-SGA mothers, statistically and clinically relevant differences were found in this group, including correlation between crematocrit and maternal nutritional status, alerting to the greater vulnerability of preterm infants small for gestational age.

The strengths of this study were the large sample size and the stratification of groups, which allowed to evaluate the effect of gestational age and fetal growth. This study brings about new insights that reinforce the benefits and value of breastfeeding for all newborns, whether they are premature and/or small for gestational age. Further research is recommended to increase knowledge about colostrum lipid composition.

Results drive us to conclude that the fat content and estimated energy value of colostrum do not change according to gestational age and fetal growth.
